# A step-by-step approach to patients leaving against medical advice (AMA) in the emergency department

**DOI:** 10.1007/s43678-022-00385-y

**Published:** 2022-10-31

**Authors:** Gabrielle Trépanier, Guylaine Laguë, Marie Victoria Dorimain

**Affiliations:** Department of Family Medicine and Emergency Medicine, Faculty of Medicine and Health Sciences, University of Sherbrooke, 3001 12e Avenue Nord, Sherbrooke, QC J1H 5N4 Canada

**Keywords:** Leaving against medical advice (AMA), Emergency medicine, Vulnerable population, Patient discharge, Refusal of care, Départ contre avis médical, Médecine d’urgence, Population vulnérable, Refus de soins, Urgentologue

## Abstract

**Objectives:**

Patients leaving against medical advice (AMA) can be distressing for emergency physicians trying to navigate the medical, social, psychological, and legal ramifications of the situation in a fast-paced and chaotic environment. To guide physicians in fulfilling their obligation of care, we aimed to synthesize the best approaches to patients leaving AMA.

**Methods:**

We conducted a scoping review across various fields of work, research context and methodology to synthesize the most relevant strategies for emergency physicians attending patients leaving AMA. We searched Medline, CINAHL, PSYCHO Legal Source, PsycINFO, PsycEXTRA, Psychological and Behavioural Sciences collection, SocIndex and Scopus. Search strategies included controlled vocabulary (i.e., MESH) and keywords relevant to the subject chosen by a team of four people, including two specialized librarians.

**Results:**

The literature review included 34 relevant papers about approaches to patients leaving AMA: 8 case presentations, 4 ethical case analyses, 10 legal letters, 4 reviews and 8 original studies. The main identified strategies were prioritizing a patient-centered approach, proposing alternative discharge and reducing harm while properly documenting the encounter.

**Conclusion:**

A systematic approach to patients leaving AMA could help improve patient care, support physicians and decrease stigmatization of this population. We advocate that emergency physicians should receive training on how to approach patients leaving AMA to limit the impact on this vulnerable population.

**Supplementary Information:**

The online version contains supplementary material available at 10.1007/s43678-022-00385-y.

## Résumé

**Objectifs:**

Les patients qui quittent contre avis médical peuvent être angoissants pour les médecins d’urgence tentant de naviguer les ramifications médicales, sociales, psychologiques et juridiques dans un environnement chaotique et au rythme effrené. Afin de guider ces médecins dans l’accomplissement de leur obligation de soins, nous avons cherché à synthétiser les meilleures approches dans la littérature face à cette situation.

**Méthodes:**

Nous avons réalisé une revue de la littérature de type « scoping review» dans une grande variété de domaines de travail, de contextes de recherche et de méthodologies afin de synthétiser les stratégies les plus pertinentes visant à guider les médecins d’urgence faisant face à un départ contre avis médical. Cette recherche a été effectuée dans plusieurs banques de données: Medline, CINAHL, PSYCHO Legal, Source, PsycINFO, PsycEXTRA, psychological and Behavioral sciences collection, SocIndex et Scopus. Les stratégies de recherche comprenaient un vocabulaire contrôlé (soit les MESH) et des mots clés pertinents au sujet choisis par une équipe de quatre personnes, dont une bibliothécaire universitaire spécialisée.

**Résultats:**

Cette revue de littérature a identifié 34 études pertinentes sur les stratégies pour les patients quittant contre avis médical: huit présentations de cas, quatre analyses éthiques, dix lettres d’opinion d’experts juridiques, huit recherches originales et quatre revues de littérature. Les principales stratégies identifiées proposent de préconiser une approche centrée sur le patient, de proposer un congé alternatif et de diminuer les impacts pour le patient tout en documentant chacune des étapes de la démarche.

**Conclusion:**

Une approche systématique des patients qui quittent contre avis médical pourrait aider la pratique, soutenir les médecins et réduire la stigmatisation de cette patientèle. Nous suggérons que les médecins d’urgence reçoivent une formation visant la prise en charge de ces patients afin de limiter les impacts sur cette population vulnérable.

## Clinician’s capsule

**Table:** 

***What is known about the topic?***
Patients leaving AMA represent a frequent but complex situation that concerns vulnerable populations, and emergency physicians often feel distraught.
***What did this study ask?***
What are the best approaches found in literature regarding patients leaving against medical advice in the emergency department?
***What did this study find?***
Physicians should prioritize a patient-centred approach, propose an alternative discharge and reduce harm while adequately documenting the interaction.
***Why does this study matter to clinicians?***
A simple step-by-step approach could help emergency physicians navigate the complex interaction arising from patients leaving AMA while reducing stigmatization of patients.

## Introduction

Emergency physicians struggle daily with the responsibility and potential implications of patients leaving against medical advice (AMA) [1]. Such patients represent up to 1 in 50 hospital discharges [2] and are associated with higher healthcare costs [3, 4]. They are indeed at higher risk of negative outcomes—such as readmission rates up to four times higher than usual [5, 6], and higher morbidity and mortality rates [7, 8]. Moreover, vulnerable and stigmatized patients (including patients with substance abuse, low income or education, and mental health problems) are overly represented in this population [5, 6, 8–10]. Patients' frequent reasons for leaving may include disagreement with the treatment plan, long emergency department waiting time, perceived improvement of their condition, family obligations, financial constraints or dissatisfaction with the services received [7]. During the pandemic, patients also left because of fear of contracting COVID-19 [11].

While patients leaving AMA is a well-documented problem, little information is known about how physicians should handle these situations in the context of an emergency department. A constellation of issues is associated with leaving AMA, including the risk of stigmatization of patients and legal retaliation [12–15]. Patients leaving AMA can be fairly frustrating to doctors and are a source of distress, as physicians tend to take the refusal personally and feel powerless or even guilty [12–15]. The issue of patients leaving AMA is also time sensitive, which adds pressure on the patient–physician communication in a crowded emergency department [15, 16]. The discharge of patients as AMA is not routinely taught in medical schools, leading to physicians being ill equipped to address the situation [14].

Emergency physicians need guidelines suited to their context to address these issues arising from patients leaving AMA in the emergency department. Therefore, this project aims to address this gap and synthesize the best practices surrounding the discharge of patients leaving against medical advice in emergency departments.

## Methods

The review aimed to synthesize the most relevant concepts to guide emergency physicians attending patients leaving AMA. A scoping review of the literature was conducted in April 2021 (updated in January 2022), in accordance with the PRISMA-ScR methodology [17]. The choice was based on the purpose of a scoping review to "identify the type of available evidence in a given field" and "identify key characteristics or factors related to a concept," as described by Munn [18], and supported by Grant and Booth's typology of reviews [19]. The two principal authors are an emergency physician with a master’s degree in health law and policy, and a family physician with a Black Belt Certification in Quality Improvement (CQI).

Our research strategies included controlled vocabulary (i.e., MESH) and keywords relevant to the subject chosen by a team of four people: two medical librarians and the three authors. The research itself was conducted in multiple databases. We searched through Medline, CINAHL, PSYCHO Legal Source, PsycINFO, PsycEXTRA, Psychological and Behavioural Sciences collection, SocIndex and Scopus databases. The search was limited to the last 10 years to reflect best the current clinical practices with patients leaving AMA. The search strategies and keywords are available in the additional materials section (Online Appendix I). We excluded patients who left without being seen or eloped since we were interested in patients under the doctor's care. We excluded studies about pediatric patients, studies in underdevelopped countries, studies not conducted in a hospital and articles not written in English or French.

The two leading reviewers established the eligibility criteria. Articles describing strategies or approaches for patients leaving AMA were included. Following review procedures, we accepted variability in study designs and genres like legal letters or ethical analysis. We excluded articles based on personal views or personal essays to avoid personal experiences.

**Fig. 1:**
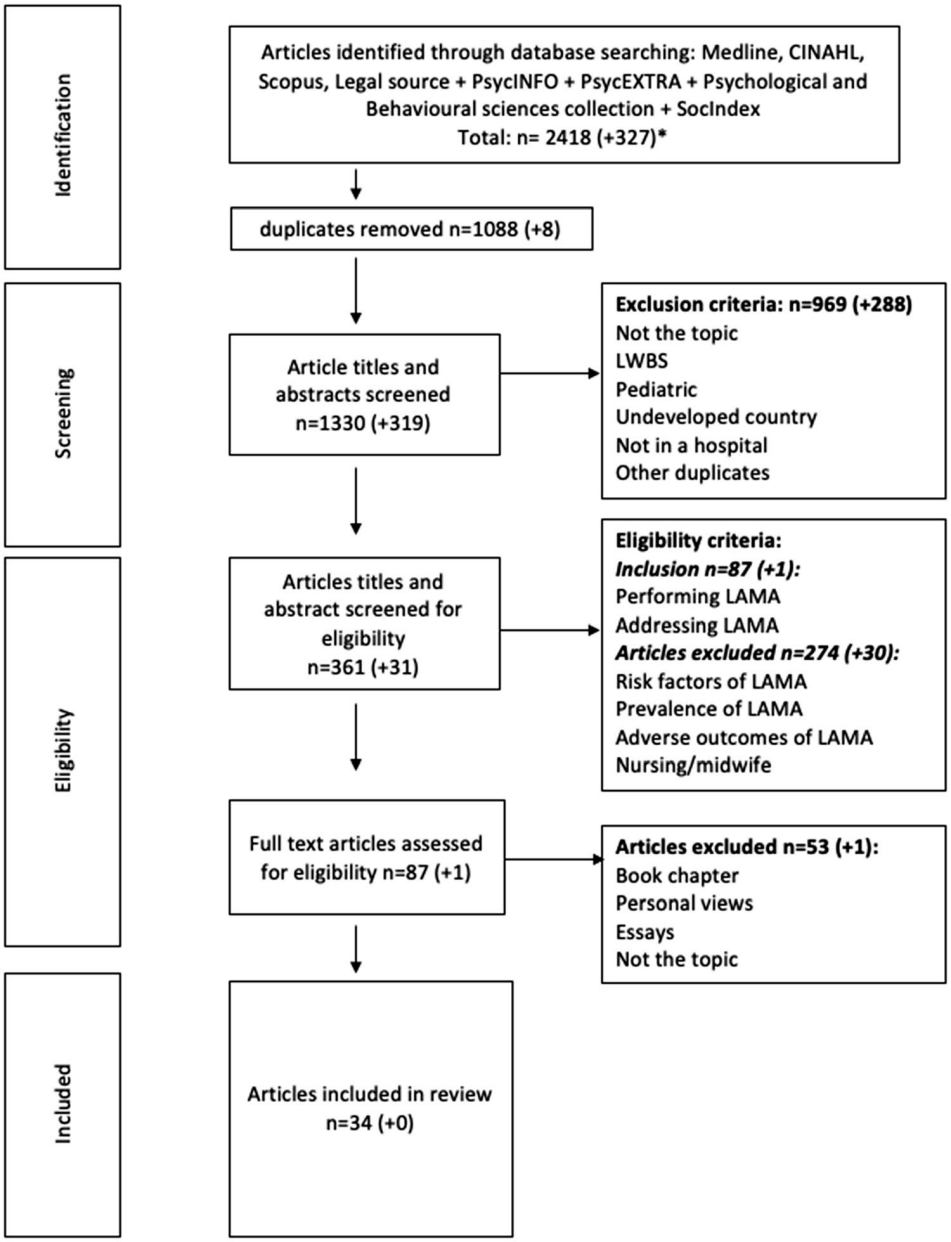
PRISMA flowchart of the selection process done in April 2021. *The numbers in brackets signify the additional results of the repeat of the search in January 2022

A total of 88 articles went through a full-text assessment. The two principal authors read all the articles, and dissidence about inclusion was discussed with the third author. Following this process, 34 articles were included in the final review (see Fig. 1 PRISMA-ScR). The two reviewers independently extracted the key characteristics and detailed information about strategies or approach for patients leaving AMA. An Excel table sheet was used for the data charting. We used an iterative process between the two reviewers to identify the key themes as they emerged from our charting [17].

## Results

### Search results and characteristics

**Fig. 2:**
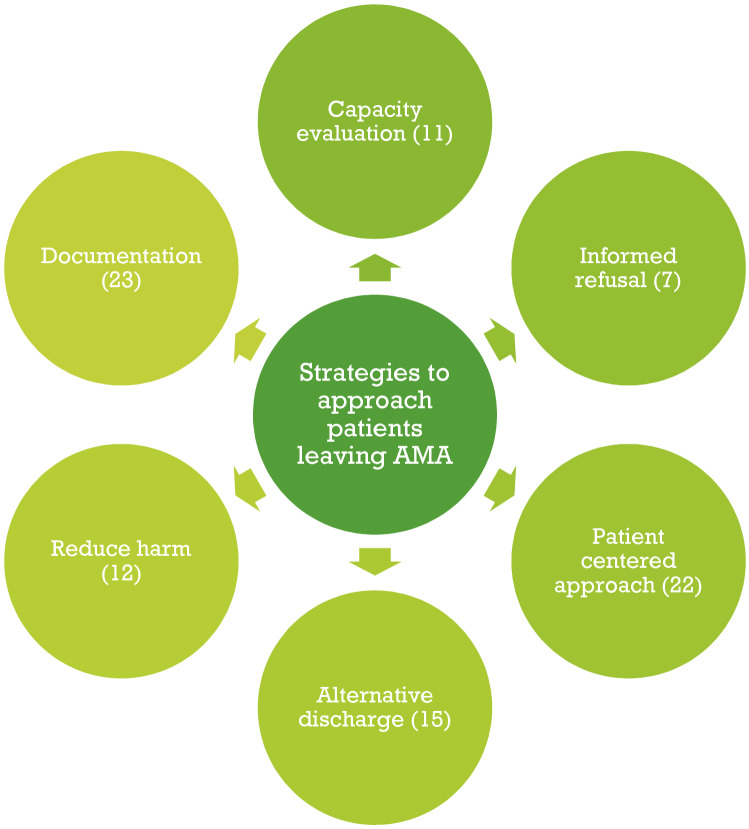
Themes identified with a number of studies relevant to the theme

We retrieved 34 papers related to strategies to approach patients leaving AMA: 8 case presentations, 4 ethical case analyses, 10 legal letters, 4 reviews and 8 original studies. The main findings are detailed in Table 1. Overall, the articles were limited by the quality of the methodological approach, but were rich in inputs from the patients' and physicians' perspectives due to ethics and legal case analysis and presentation. The two reviewers extracted 142 citations and excerpts that were regrouped under six main themes: patient-centered approach, capacity evaluation, informed refusal, alternative discharge, harm reduction and documentation (see Fig. 2). The principal findings are presented in a narrative format.

**Table 1:** Presentations of studies and principal themes (attached file)

Clinical case presentation and ethical case analyses					
Authors (year of publication)	Title	Country of publication	Domains/themes	Strengths	Weaknesses
Alfandre (2013) [35]	Reconsidering against medical advice discharges: embracing patient-centeredness to promote high quality care and a renewed research agenda	USA	Alternative discharge; documentation	Can be published quickly Provides very detailed information Explore ethical reflection on specific issues	May include authors/researchers bias Cannot always be generalized to the broader population Very low level of evidence
Alfandre et al. (2017) [33]	Against Medical Advice Discharges	USA	alternative discharge; harm reduction; documentation		
Brzezinski et al. (2017) [27]	Discharge against Medical Advice in Surgical Patients with Posttraumatic Stress Disorder: A Case Report Series Illustrating Unique Challenges	USA	Patient-centered approach		
Clark et al. (2014) [36]	*Ethics seminars: a best-practice approach to navigating the against-medical-advice discharge	USA	Patient-centered approach; alternative discharge; harm reduction; documentation; capacity		
Marco et al. (2017) [30]	*Refusal of Emergency Medical Treatment: Case Studies and Ethical Foundations	USA	Patient-centered approach; alternative discharge; informed refusal; harm reduction; documentation; capacity		
Mukherjee (2015) [21]	Discharge Decisions and the Dignity of Risk	USA	Patient-centered approach		
Nelson et al. (2014) [16]	Responding to the refusal of care in the emergency department	USA	Capacity		
Taylor and Geppert (2011) [26]	You Say "Yes," I Say "No," You Say "Goodbye," and I Say "Hello"	USA	Patient-centered approach; informed refusal		
Rudofker and Gottenborg (2019) [25]	Avoiding Hospital Discharge Against Medical Advice: A Teachable Moment	USA	Alternative discharge; documentation		
Shuman and Barnosky (2012) [39]	Exploring the limits of autonomy	USA	Alternative discharge; patient-centered approach		
Stern et al. (2011) [23]	Prior discharges against medical advice and withdrawal of consent: what they can teach us about patient management	USA	Patient-centered approach		
West (2020) [37]	What Is an Ethically Informed Approach to Managing Patient Safety Risk During Discharge Planning?	USA	Alternative discharge		

Legal letters					
Authors (year of publication)	Title	Country of publication	Domains/themes	Strengths	Weaknesses
Ahc (2018) [13]	Patients Leaving Against Medical Advice Create Liability Risk	USA	Patient-centered approach	Provides very detailed information	Cannot always be generalized to the broader population (legislation differs from countries)
Ahc (2019) [38]	Legal Exposure for ED When Overdose Patients Refuse Care	USA	Alternative discharge; documentation; capacity	Explore legal aspects on very specific issues	Expert opinions are very low level of evidence
Ahc (2019) [12]	Patients Leaving AMA Require Good Communication to Avoid Liability	USA	Patient-centered approach; documentation; capacity		
Ahc (2019) [45]	Patients Leaving AMA: Signed Forms Alone Are Not Sufficient Malpractice Defense	USA	Harm reduction		
Broida et al. (2017) [46]	Does ED Chart leave AMA patient free to claim, ‘If Only I’d Known the Risks?’	USA	Patient-centered approach; alternative discharge; informed refusal; harm reduction, documentation; capacity		
Derse and Greenfleder (2012) [34]	"How Could You Have Let This Person Leave Your ED?"	USA	Patient-centered approach; harm reduction; documentation; capacity		
Gallagher et al. (2016) [32]	Patient’s Signature on AMA Form Won't Stop Successful Lawsuit	USA	Documentation		
Klauer (2014) [47]	Step in Before Patient Leaves ED Unhappy: Stop Possible Suit	USA	Documentation		
Morley (2020) [22]	Reducing risks of patients leaving against medical advice	USA	Informed refusal		
Patient Safety Institute (2016) [43]	When the patient makes a poor choice, will a signed AMA form protect me?	USA	Patient-centered approach; documentation		

Original research						
Authors (year of publication)	Title	Country of publication	Type of research	Domains/themes	Strengths	Weaknesses
Brenner et al. (2016) [28]	Against Medical Advice: A Survey of ED Clinicians' Rationale for Use	USA	Retrospective cohort study	Patient-centered approach; documentation	Collects factual information about an existing phenomenon Allows to appreciate current practices	Important recall bias: small number of physicians included in the study
Edwards et al. (2013) [41]	Discharge against medical advice: how often do we intervene?	USA	Retrospective cohort study	Patient-centered approach; harm reduction; documentation		Single institution: possibility to miss some information that may not be recorded in charts
Lekas et al. (2016) [20]	The role of patient-provider interactions: Using an accounts framework to explain hospital discharges against medical advice	USA	Retrospective cohort study	Patient-centered approach; alternative discharge; harm reduction; documentation		Small sample of 33 HIV infected patients Limits the scope of this study’s applicability
Machin et al. (2018) [15]	An Alternative View of Self-Discharge Against Medical Advice: An Opportunity to Demonstrate Empathy, Empowerment, and Care	UK	Semi-structured in depth interviews	Patient-centered approach; alternative discharge; informed refusal; harm reduction; documentation	Allows a thorough understanding of the phenomenon	Small sample size Limited demographic details, and Self-selecting participants
Marco et al. (2021) [24]	Refusal of emergency medical care: An analysis of patients who left without being seen, eloped, and left against medical advice	USA	Prospective study	Patient-centered approach	Description of a specific population	Only descriptive data No intervention
Schaefer and Monico (2013) [44]	Documentation proficiency of patients who leave the emergency department against medical advice	USA	Retrospective cohort study	Documentation	Collects factual information about an existing phenomenon Allows to appreciate current practices	Limited data by using (maybe incomplete) charts Single center
Stearns et al. (2017) [40]	Discharges Against Medical Advice at a County Hospital: Provider Perceptions and Practice	USA	Mixed methods cross sectional study	Patient-centered approach; documentation		Limited to a single center Might not be generalizable to other settings Retrospective chart review limited to information documented in the medical record
Tummalapalli et al. (2020) [42]	Physician Practices in Against Medical Advice Discharges	USA	Cross sectional study	Alternative discharge; informed refusal; documentation		Limited to a single center No evaluation of the physicians’ characteristics

Review						
Authors (year of publication)	Title	Country of publication	Type of research	Domains/themes	Strengths	Weaknesses
Albayati et al. (2021) [1]	Why Do Patients Leave against Medical Advice? Reasons, Consequences, Prevention, and Interventions	USA	Literature review	Patient-centered approach; alternative discharge; harm reduction; informed refusal; documentation; capacity	Identifies what has been accomplished Summation	Lack an explicit intent to maximize scope or analyze data Possible bias by selecting literature supporting their views Method not always described in these reviews
Holmes et al. (2021) [14]	Against Medical Advice Discharge: A Narrative Review and Recommendations for a Systematic Approach	USA	Narrative review	Patient-centered approach; alternative discharge, documentation, capacity		
Kahle et al. (2015) [29]	Discharges Against Medical Advice: Considerations for the Hospitalist and the Patient	USA	Clinical guidelines review	Patient-centered approach; alternative discharge; harm reduction; documentation; capacity		
Levy et al. (2012) [31]	The importance of a proper against-medical-advice (AMA) discharge: how signing out AMA may create significant liability protection for providers	USA	Legal narrative review	Patient-centered approach; harm reduction; documentation; capacity

### Approach strategies for patients leaving AMA

#### Patient-centered approach

A strategic way for physicians to approach patients leaving AMA is to position them at the center of care by actively listening to their concerns, thus increasing the quality of communication [15, 20, 21]. Physicians must aim without prejudice or bias to identify why patients wish to leave AMA [1, 12, 22–24]. Correctly identifying the reason for departure is essential to set a base for the patient–physician discussion. It is an opportunity to acknowledge their concerns and alleviate some or express empathy [15, 25–27]. Involving a multidisciplinary team or asking for an early psychiatric consult may be helpful [1, 28].

#### Capacity evaluation

An important reminder is that patients leaving AMA must have decision capacity. Although determination of capacity is beyond the scope of this review, some particularities apply specifically to the AMA patients, and, physicians must keep them in mind. All patients have presumption of capacity [29] and can decide to leave, even if it is not in their best interest [12]. Also, a patient's capacity to consent or refuse treatment is dynamic and task specific [16, 30], which makes capacity evaluation challenging [31] with the time constraints in the emergency department [16].

#### Informed refusal

When a patient signifies the desire to leave AMA, emergency physicians must explain the benefits of completing the treatment and also explain the risks associated with leaving [32]. Although it is impossible to list all possible risks, physicians must emphasize possible complications or worsening of the patient's medical condition and name the risk for any permanent disability, as well as the risk of death, if applicable [30, 32]. Emergency physicians must remain cautious, validate patients' understanding of what they are refusing and allow enough time for patients to ask questions [33]. In the context of an emergency department, physicians may not have the option to get an informed refusal [34].

#### The alternative discharge

Physicians should support patients in their choice of treatment, regardless of the initially recommended option. Understanding a patient's goal for treatment helps the patient–physician alliance [20, 33, 35]. When patients refuse the recommended treatment plan, engaging them in decision-making can help elaborate an alternative discharge plan [33, 36]. Providing an alternative discharge, while suboptimal, is not necessarily substandard [1, 25, 33, 37, 38] and can be adapted to the patient's situation. Also, refusing to provide an alternative discharge to patients and simply accepting that the patient leaves AMA has even been described as abandonment by some ethicists [36]. The nomenclature itself, "AMA discharge", can be seen as paternalistic, which may unwillingly promote stigmatization of patients. Therefore more neutral terms such as "premature discharge" or "alternative discharge" have been proposed [15].

#### Harm reduction

When AMA departure is inevitable, all efforts should be geared toward organizing the safest discharge scenario and reducing harm [15]. Physicians are ethically obligated to arrange follow-up and outpatient treatment [1, 29, 36]. They may also, for example, need to assist the patient in leaving the hospital by arranging transportation for example [39]. They should go through the discharge process like any other discharge: give instructions, inform patients of any investigations that may have been conducted before discharge and provide the relevant prescriptions [30, 32, 37]. Even though it has been shown that up to 94% of attending physicians agree that AMA patients should receive medication and follow-up arrangements [40], studies indicate that only a quarter of these patients do receive a prescription and that only a third of them are discharged with a follow-up plan [40–42].

In addition, as one out of five patients who left AMA feel reluctant to return to the emergency department because they thought they angered staff [13, 20, 32], physicians must refrain from aggressively attempting to convince them to stay and inform them to consult again, if necessary. The physicians are also responsible for the follow-up of any pending results and should verify how to contact patients if necessary [13].

#### Documentation

Proper documentation of AMA (see Table 2) is quite exhaustive. Still, it should at least provide evidence of a patient's capacity and informed refusal [34, 43]. Exhaustive documentation was found in only 4% of the charts in a small study [44]. Capacity is documented in about 20–60% of files [30, 40–42, 44], and a discussion about risks is documented in about 60–70% of files [30, 42]. The primary reason to designate the discharge as AMA is the fear of legal liability [33]. However, the documentation of the discussion with patients is essential for the physicians' legal protection. Labeling the discharge as AMA does not confer legal protection [13, 32].

**Table 2:** Comprehensive documentation includes [1, 25, 29, 38, 43, 45–47]

Capacity assessment
Description of the interaction with the patient
Physician's concerns
Extent and limitations of the ED evaluation
Explications of risk and benefits
Alternatives discussed
Use of a language the patient understands
Patient opportunity to ask question
Evidence the patient/family understands
Patient was informed to return to the ED at anytime
Notification to the primary physician
Evidence of a harm reduction approach

It is debated whether an AMA form should be used or not. Getting it signed should never distract physicians from fulfilling their obligation of care and supporting patients through the AMA discharge process [33]. The advantage of using an AMA form is to bolster documentation, facilitate discussion with patients or ease the documentation process [28]. A small study found that a standard form increased capacity documentation from 0 to 80% and patients' signatures from 58 to 80% [31]. The signature rates vary in different studies from 58 to 85% [28, 31]. If a patient refuses to sign the AMA form, the issue should not be pressed. It is possible to obtain the signature of a witness of the conversation (nurse, family member, etc.) [30, 34]. Experts agree that a signed form alone is not enough to ensure legal protection and to attest capacity [15, 25, 29, 30, 32, 35, 40, 43, 45].

## Discussion

### Interpretation

This study provides an extensive review of different fields of research. Insight from ethicists, lawyers, quality experts and physicians from different domains helped to grasp a better understanding of how to approach patients leaving AMA. This literature review identified different strategies for patients leaving AMA and allowed us to adapt an AMA discharge approach to emergency patients. Since AMA discharge can reflect a conflict between a patient's and physician's perspectives, developing a practical approach to patients leaving AMA in the emergency department is essential to negotiate the problem's medical, ethical, social and legal aspects. We think that this proposed step-by-step approach could help emergency physicians attend to patients leaving AMA and meet their obligation of care (Table 3).

**Table 3:** A step-by-step approach to patients leaving AMA

Approach to patients who want to leave AMA^a^			
1	Try a patient-centered approach	Seek why the patient leaves AMA without assuming or stigmatizing the patient [1, 12, 22] Address the patient's concerns and needs [15] Think of a multidisciplinary approach, psychiatric consult [1, 28]	Comprehensive documentation
2	Propose an alternative plan	Support patients on their treatment goals [20, 33, 35] Do not judge why the patient is leaving [14, 20] Remember, a suboptimal plan is better than no plan and is not necessarily substandard [33, 38]	
33	Reduce harm	Do an informed refusal if applicable [30, 32, 34] Give instructions on the testing results and provide prescriptions and follow-up [30, 32, 37] Advise the patient to return at any time if needed [13, 32]	

^a^Patients must have decision-making capacity

### Step-by-step approach

Once the patient is deemed competent, emergency physicians should first determine if they genuinely want to leave AMA or if it entails for something else. Physicians should prioritize a patient-centered approach [1, 12, 15, 22, 28], to understand patients' perspectives to avoid stigmatization and find a collaborative way of care. If the patient is determined to leave, physicians should not judge the veracity or legitimacy of their reason to leave. They should propose alternative discharges [14, 20, 33, 35, 38], even if they are suboptimal.

If the patient refuses all discharge alternatives, the emergency physicians should try to obtain an informed refusal [26, 30, 32, 33, 46] and adopt a harm reduction approach [13, 15, 30, 32, 37]. Physicians should provide prescriptions, follow-up and continuity of care when applicable. Experts suggest approaching all discharges similarly and avoiding labeling AMA discharge to prevent stigmatization [15].

Finally, through this discharge process, all relevant information should be carefully documented in the patient's chart [15, 25, 29, 30, 32, 35, 40, 43, 45]. However, physicians are not obligated to use a formal AMA form and should not pressure patients into singing it.

### Previous studies

Our study stands out by adopting a more practical approach to patients leaving AMA. We present a more concise and direct clinical approach that does not solely focus on reducing legal risk and emphasize patient’s perspective to provide better care. Previous studies, like the two other review done in 2021[1, 14], on how to approach patients leaving AMA are quite exhaustive and include multiple steps [1, 14, 29, 31], which is not realistic for the frequently time-sensitive, chaotic clinical conditions and time pressure in which emergency physicians work. We present a more concise and direct clinical approach that does not solely focus on reducing legal risk and emphasizes the patient's perspective to provide better care.

### Strengths and limitations

This review intended to extend the scope of research to other fields like ethics and law to find all possible strategies about the issue of patients leaving AMA. We worked with specific inclusion and exclusion criteria, and both reviewers independently selected eligible articles. Included studies were limited in methodological quality, as most were case presentations, ethical case analyses and legal letters. Still, they were rich in patients' and physicians' perspectives. However, since we did not do a critical appraisal, the proposed approach should be considered with caution, since scoping reviews have the "danger that the existence of studies rather than their intrinsic quality is used as the basis for conclusion" [19]. Also, legal implications and consequences can vary across borders, and we tried to be as inclusive as possible. Therefore, caution should be taken for applicability.

### Clinical and research implications

We think this step-by-step approach could help shift the medical culture by reducing bias and stigmatization. For example, it could possibly increase the usage of "alternative discharge" over "AMA discharge" in the charting vocabulary. Another example is adapting discharge processes to patients' needs and using harm reduction strategies more frequently. The next step is  to teach the step-by-step approach to physicians and medical students and implement the approach in a clinical setting  to evaluate if more alternative discharges are planned. Further research is needed to determine how to best implement this step-by-step approach in emergency settings.

## Conclusion

Patients leaving AMA is a persistent and distressing problem with medical, ethical, social and legal impacts for patients and physicians. Emergency physicians receive minimal training to address the problem and are therefore poorly equipped to face the situation. We advocate that emergency physicians should receive training on how to approach patients leaving AMA to limit the impact on this vulnerable population. A simple step-by-step approach to patients leaving AMA could support physicians, alleviate bias and reduce stigmatization of patients.

## Supplementary Information

Below is the link to the electronic supplementary material.

Supplementary file1 (DOCX 17 KB)

Supplementary file2 (PDF 517 KB)
