# Supplementary material for: A step-by-step approach to patients leaving against medical advice (AMA) in the emergency department
Source: CJEM. 2022 Oct 31;25(1):31–42. doi: 10.1007/s43678-022-00385-y (PMC9628312; doi:10.1007/s43678-022-00385-y)
Supplement: Supplementary file 1 — Supplementary file1 (DOCX 17 KB) [file 43678_2022_385_MOESM1_ESM.docx]

| Appendix1: Strategies | | |
| --- | --- | --- |
| EBSCO: Database - MEDLINE with Full Text;CINAHL Plus with Full Text;Legal  Source;APA PsycExtra;Psychology and Behavioral Sciences Collection;APA  PsycInfo;SocINDEX | S1 | TI ( leav* OR left OR discharg* OR depart* ) OR AB ( leav* OR  left OR discharg* OR depart* ) |
|  | S2 | TI ( "denial of care" OR "refusal of care" OR "refusal of  treatment" OR "refusing treatment" OR "against medical advice"  OR "treatment refusal" ) OR AB ( "denial of care" OR "refusal of  care" OR "refusal of treatment" OR "refusing treatment" OR  "against medical advice" OR "treatment refusal" ) |
|  | S3 | (MH "Patient Discharge") |
|  | S4 | (MH "Treatment Refusal") |
|  | S5 | S1 OR S3 |
|  | S6 | S2 OR S4 |
|  | S7 | S5 AND S6 |
|  | S8 | S5 AND S6 + l Limiters - Date of Publication: 2011/01/01-2021/12/31 (10 years) |
|  | ** | In the CINAHL plus full-text database the CINAHL-headings (patient discharge, early patient discharg) were also selected |
| Scopus | Search | ( ( TITLE ( leav* OR left OR discharg* OR depart* ) OR ABS ( leav* OR left  OR discharg* OR depart* ) ) ) AND ( ( TITLE ( "denial of care" OR "refusal of  care" OR "refusal of treatment" OR "refusing treatment" OR "against medical  advice" OR "treatment refusal" ) OR ABS ( "denial of care" OR "refusal of  care" OR "refusal of treatment" OR "refusing treatment" OR "against medical  advice" OR "treatment refusal" ) ) ) AND ( LIMITTO  ( PUBYEAR , 2021 ) OR LIMIT-TO ( PUBYEAR , 2020 ) OR LIMITTO  ( PUBYEAR , 2019 ) OR LIMIT-TO ( PUBYEAR , 2018 ) OR LIMITTO  ( PUBYEAR , 2017 ) OR LIMIT-TO ( PUBYEAR , 2016 ) OR LIMITTO  ( PUBYEAR , 2015 ) OR LIMIT-TO ( PUBYEAR , 2014 ) OR LIMITTO  ( PUBYEAR , 2013 ) OR LIMIT-TO ( PUBYEAR , 2012 ) OR LIMITTO  ( PUBYEAR , 2011 ) ) |
